# Susceptibility to postbiotics - enterocins of methicillin-resistant *Staphylococcus aureus* strains isolated from rabbits

**DOI:** 10.1007/s11259-024-10323-1

**Published:** 2024-02-07

**Authors:** Natália Zábolyová, Andrea Lauková, Monika Pogány Simonová

**Affiliations:** 1 Centre of Biosciences of the Slovak Academy of Sciences, Institute of Animal Physiology, Šoltésovej 4-6, Košice, 04001 Slovakia; 2 University of Veterinary Medicine and Pharmacy in Košice, Komenského 73, Košice, 04181 Slovakia

**Keywords:** Methicillin-resistance; *Staphylococcus aureus*, Enterocin, Antibiofilm effect

## Abstract

There is a major problem with the rising occurrence of highly virulent and multiply-resistant strains, including methicillin-resistant *Staphylococcus aureus* (MRSA), because of their difficult treatment. This study aimed to evaluate the antibacterial and antibiofilm effect of new enterocins (Ent) against potential pathogenic MRSA strains isolated from rabbits. Staphylococci were identified with PCR and screened for methicillin/oxacillin/cefoxitin resistance (MR) using the disk diffusion method and the PBP2’ Latex Agglutination Test Kit. Enzyme production, hemolysis, DNase activity, slime production, and biofilm formation were tested in MRSA strains. The susceptibility of MRSA to eight partially-purified enterocins (Ent) produced by *E. faecium* and *E. durans* strains was checked using agar spot tests. The antibiofilm activity of Ents was tested using a quantitative plate assay. Out of 14 MRSA, PBP testing confirmed MR in 8 strains. The majority of MRSA showed DNase activity and β-hemolysis. Slime production and moderate biofilm formation were observed in all strains. MRSA were susceptible to tested Ents (100–12,800 AU/mL; except Ent4231). The antibiofilm effect of Ents (except Ent4231) was noted in the high range (64.9–97.0%). These results indicate that enterocins offer a promising option for the prevention and treatment of bacterial infections caused by biofilm-forming MRSA.

## Introduction

*Staphylococcus aureus* is found as a commensal on the skin and nasal microbiota of healthy humans and animals. On the other hand, during the last years, methicillin-resistant (MRSA) and multidrug-resistant *S. aureus* (MDSA) were noted as major causes of hospital-, community-, and livestock-acquired infections ranging from wound infections to life-threatening septicemia and toxic shock syndrome in humans (Götz 2002) and from small skin lesions to invade subcutaneous tissue in domestic and food-producing animals, causing well-known mastitis, abscesses, and pododermatitis connected also with economic losses (Lozano et al. 2016; Bzdil et al. 2021). Because of the high capacity of pathogenic staphylococci to acquire resistance traits, therapy for these infections with antibiotics is usually ineffective. Moreover, *S. aureus* can strongly adhere to host tissues and consequently form biofilms (Merghni et al. 2016). Therefore, novel natural antimicrobial therapies are an urgent need. It has been shown that the use of probiotics (live, nonpathogenic microorganisms) and postbiotics (inanimate bacteria, cell components, metabolites or post-fermentation by-products) reduces the occurrence of pathogens in large-scale farms. Nowadays, postbiotics bring new inspiration for the modulation of gut health due to their advantages - distinctive stability, safety, and functional diversity (Aggarwal et al. 2022; Zhong et al. 2022). Bacteriocins as a biggest group of postbiotics have attracted attention as potential antimicrobial compounds to reduce or eliminate MRSA and prevent their infections and biofilm formation (Mathur et al. 2018). Bacteriocins are biologically active proteins or peptides ribosomally synthesized by several bacterial strains with antimicrobial effects against bacteria that are more or less related to the producer strains (Klaenhammer 1993). There are several groups of bacteriocins, classified according to their biochemical, genetic properties and mode of action (Nes and Holo 2000). Most studies provide the promising potential of bacteriocins regarding their antimicrobial activity, broad and narrow inhibitory spectrum against pathogens without disturbing the commensal bacterial microbiota, resistance to heat and pH variation, and low toxicity. The most studied bacteriocins are lantibiotics (both commercial – Nisin, Gallidermin, and new) regarding also their antibacterial/antibiofilm effect and therapeutic application against MDSA and MRSA (van Staden et al. 2021). However, enterocins (small, termo-stable bacteriocins produced by enterococci; Franz et al. 2007) are also characterized by strong antimicrobial activity; studies about their anti-MRSA activity, including their antibiofilm effect, are limited (Al Atya et al. 2016; Pogány Simonová et al. 2021). Although the use of bacteriocins for microbial biofilm control is a relatively new research field, the achieved results are promising, and new testing is required to expand the knowledge regarding the antibiofilm activity of enterocins.

Therefore, this study aims to evaluate (in vitro) the antibacterial and antibiofilm effects of new, not commercialized enterocins against biofilm-forming MRSA isolated from rabbits.

## Materials and methods

### Isolation and microbial procedures

Staphylococci were isolated from fecal, cecal, and meat samples (142) from rabbits of both sexes and various ages (from two to four months), breed on 16 different farms in West Slovakia. Sampling and animal handling followed the Guide for the Care of Animals accepted by the Ethic Committee at the Institute of Animal Physiology, Centre of Biosciences of the Slovak Academy of Sciences (IAP CBs SAS), Košice, Slovakia, and by the Slovak Veterinary and Food Administration. Samples (1 g) were treated using the standard microbial dilution method according to ISO; they were stirred (1:9) in Ringer solution (pH 7.0, Merck, Darmstadt, Germany); appropriate dilutions were spread on Baird-Parker agar (Difco Laboratories, Franklin Lakes, NJ, USA) supplemented with egg yolk tellurite solution (ISO 21527-1, Difco), and incubated at 37 °C for 24–48 h. Representative colonies (3–5 per sample) were picked up, checked for their purity, and submitted for identification for *Staphylococcus aureus* strains previously described by Simonová et al. (2007). Among 110 staphylococcal isolates (65 from feces, 9 from cecum, and 39 from meat) specified by PCR, only 14 (5 from feces and 9 from meat) were allotted to the *S. aureus* species.

### Disc diffusion test to detect methicillin, oxacillin and cefoxitin and detection of penicillin-binding protein (PBP)2´

The antibiotic phenotypic profile of *S. aureus* strains was tested using the agar disk diffusion method (Clinical Laboratory Standard Institute guideline, CLSI 2022). Strains were cultivated in Brain Heart Infusion (BHI; Difco Laboratories, Detroit, MI, USA) overnight (A600 up to 0.8), then 100 µL volumes were spread on Mueller Hinton agar (Difco) and the appropriate antibiotic disks were applied. The inoculum corresponded with 0.5 MacFarland. The following antibiotics were tested: oxacillin (Oxa; 5 µg), methicillin (Met; 10 µg) and cefoxitin (Fox; 30 µg) supplied by Oxoid (Basingstoke, Hampshire, UK) to select and confirmed the methicillin-resistant staphylococcal (MRS) isolates. Plates were incubated at 37 °C for 24 h and evaluated as susceptible or resistant according to the recommendation provided by the antibiotic disk suppliers and CLSI (2022). The control strain was the *S. aureus* BD44 (CBs SAS IAP, Košice, Slovakia).

Based on the results from disk-diffusion testing, *S. aureus* strains were further tested to confirm their resistance to methicillin. The PBP2´ Latex Agglutination Test Kit (Oxoid) was used to test colonies grown for 24 h according to the manufacturer´s instructions. Quality control was done with *S. aureus* ATCC43300. Based on these positive results, eight MRSA strains were selected and tested for other activities.

### Enzyme production, nuclease activity, and hemolysis of selected strains

Selected MRSA strains (8) were characterized for their enzymatic activity using the commercial API ZYM strips (Biomérieux, France) following the manufacturer´s recommendations to detect the presence of bacterial enzymes. Enzymes evaluated were: alkalic phosphatase, esterase (C4), esterase lipase (C8), lipase (C14), leucin-arylamidase, valin arylamidase, cystin arylamidase, trypsin, α-chymotrypsin, acidicphosphatase, Naftol-AS-BI-phosphohydrolase, α-galactosidase, β-galactosidase, β-glucuronidase, α-glucosidase, β-glucosidase, N-acetyl-β-glucosamonidase, α-manosidase, α-fucosidase. Inocula (65 µL) of McFarland Standard one suspensions were pipetted into each well of the kit. Enzymatic activities were evaluated after 4 h of incubation at 37 °C and after the addition of Zym A and Zym B reagents. Color intensity values from 0 to 5 and their relevant value in nanomoles were assigned for each reaction according to the color chart supplied with the kit.

To determine nuclease activity, each strain was inoculated onto the surface of DNase agar (Oxoid) and incubated for 24 h at 37 °C. After flooding and acidifying the medium with 1 N HCl, the DNA precipitated out, and the medium became turbid with clear zones around DNase-positive colonies *S. aureus* SA4 from a dog (isolated in our laboratory by Dr. Strompfová) was used as a positive control.

Hemolysis was detected by streaking the cultures onto BHI agar (Difco) and supplementing with 5% defibrinated sheep blood. Plates were incubated at 37 °C for 24 h in an incubator. The presence or absence of clear zones around the colonies was interpreted as α- and β-hemolysis, respectively, while γ-hemolysis indicated negative strains (Semedo et al. 2003).

### Biofilm formation by qualitative and quantitative methods

To test biofilm formation in identified staphylococci, the qualitative phenotypic method with Congo red agar (CRA) was used, according to Freeman et al. (1989). The cultivation medium was composed of Brain Heart infusion (Difco, 37 g/l) enriched with sucrose (36 g/l; Slavus a.s., Bratislava, Slovakia), pure agar (30 g/l; Difco) and Congo red dye (0.8 g/l, Merck, Germany). The medium was autoclaved at 121 °C for 15 min. Plates of the medium were inoculated with the tested staphylococcal strains and incubated at 37 °C for 24 h. A positive result was indicated by black colonies with a dry crystalline consistency; strains without biofilm production usually remained pink. The color was also checked after 48 and 72 h.

A quantitative plate assay was also used to test the biofilm formation ability of MRSA strains, according to Chaieb et al. (2007). One colony of each strain grown on BHI agar overnight at 37 °C (Difco) was transferred into 5 mL of Ringer solution (pH 7.0, 0.75% w/v) to obtain a suspension corresponding to 1 × 10^8^ cfu/mL. A 100 µL aliquot from that dilution was transferred into 10 mL of BHI broth (Difco). A 200 µL volume of the dilution was inoculated into polystyrene microtiter plate wells (Greiner ELISA 12 Well Strips, 350 µL, flat bottom, Frickenhausen GmbH, Germany) and incubated for 24 h at 37 ◦C. The biofilm that formed in the microtiter plate wells was washed twice with 200 µL of deionized water and dried at 25 ◦C for 40 min. The remaining attached bacteria were stained for 30 min at 25 ◦C with 200 µL 0.1% (m/v) crystal violet in deionized water. The dye solution was aspirated away, and the wells were washed twice with 200 µL of deionized water. After the water removal, the plate was dried for 30 min at 25 ◦C, and the dye bound to the adherent biofilm was extracted with 200 µL of 95% ethanol. A 150 µL aliquot was transferred from each well into a new microplate well for absorbance (A) at 570 nm using an Apollo 11 Absorbance Microplate reader LB 913 (Apollo, Berthold Technologies, Oak Ridge, TN, USA). Each strain and condition were tested in two independent tests with 12 replicates. Sterile BHI was included in each analysis as a negative control. *Streptococcus equi* subsp. *zooepidemicus* CCM 7316 was used as a positive control (kindly provided by Eva Styková, University of Veterinary Medicine and Pharmacy, Košice, Slovakia). Biofilm formation was classified as highly positive (OD570 ≥ 1), low-grade positive (0.1 ≤ OD570 < 1), or negative (OD570 < 0.1), according to Chaieb et al. (2007). For classification, we used the average OD value and cut-off value (ODc; defined as 3 standard deviations (SD) above the mean OD of the negative control). The final OD value of a tested strain is expressed as the average OD value of the strain reduced by the ODc value. For interpretation of the results, strains were divided into the following categories: non-biofilm producer (OD ≤ ODc), weak biofilm (ODc < OD ≤ 2xODc), moderate (2xODc < OD ≤ 4xODc), and strong biofilm (4xODdc < OD), as described by Stepanović et al. (2007).

### Antibacterial and antibiofilm effects of partially purified enterocins against selected MRSA strains

Eight partially purified enterocins (Ent) were used in the testing. Most of them are produced by our *Enterococcus faecium* strains of different origins (four registered with the CCM, Brno, Czech Republic): EntA(P)/EK13, produced by *E. faecium* EK13/CCM7419 environmental strain (Mareková et al. 2003); EntM/AL41, produced by *E. faecium* AL41/CCM8558 environmental strain (Mareková et al. 2007); Ent4231, produced by ruminal strain *E. faecium* CCM4231 (Lauková et al. 1993); Ent7420, produced by rabbit-derived strain *E. faecium* EF2019/CCM7420 (Pogány Simonová et al. 2009); Ent55, produced by chicken-derived *E. faecium* EF55 (Strompfová and Lauková 2007); Ent9296, produced by silage strain *E. faecium* EF9296 (Marciňáková et al. 2008); Ent412, produced by *E. faecium* EF412 (unpublished data); DurED26E/7, produced by *E. durans* ED26E/7 (isolate from ewe lump cheese; Lauková et al. 2012). Enterocins were prepared using the following procedure: a 16 h culture (300 mL of producer strains) in De Man-Rogosa-Sharp (Merck, Germany) or Todd-Hewit (Difco) broth was centrifuged for 30 min at 10,000 × g to remove the cells. After adjusting the supernatant to pH 5.0 (5.5 for EntM/AL41), ammonium sulfate was added to the supernatants to obtain 40% (w/v) saturation and 80% (w/v) for ED26E/7. The mixture was gently stirred at the appropriate temperatures for different lengths of time depending on the enterocin-producing strain: 4 °C for 2 h (EK13/CCM7419, EF9296), 4 h (EF2019/CCM7420, EF412) and 24 h (EF55, CCM4231, ED26E/7), and at 21 °C for 1 h (AL41/CCM8558). After centrifugation at 10,000 × g for 30 min, the resulting pellet was resuspended in 10 mmol/L of sodium phosphate buffer (pH 6.5).

Antibacterial/inhibition activity was determined using the agar spot test (De Vuyst et al. 1996). Briefly, BHI supplemented with 1.5% agar (BHIA; Difco) was used for the bottom layer. For the overlay, 0.7% BHIA enriched with 200 µL of the indicator culture strain was used (A600 up to 1.0; 1.0 × 10^8^ CFU/mL). Bacteriocin dilution (100 AU/mL; 10 µL) of all Ents was dropped on the surface of soft agar with each tested staphylococcal strain, and after incubation (37 °C for 18 h), clear inhibition zones around the doses of diluted bacteriocins were read. Inhibition activity was expressed in arbitrary units per milliliter (AU/mL) as the reciprocal of the highest two-fold dilution of bacteriocins, demonstrating complete growth inhibition of the tested strain. Tests were performed twice. The principal indicator, the fecal *Enterococcus avium* EA5 strain (our isolate from piglet), was used as a positive control; its inhibition activity reached up to 102,400 AU/mL.

To determine the antibiofilm effect of the tested Ents, we used the quantitative plate assay. MRSA strains were precultured in BHI medium overnight (1.0 × 10^8^ CFU/mL) and diluted 1,000-fold with BHI medium. Aliquots (180 µl) of each bacterial suspension and 20 µL of each Ent (concentration of 12,800 AU/mL) were added to the wells of a 96-well flat-bottomed polystyrene plate and incubated for 24 h at 37 °C. After incubation, the solution was discarded, and each well was washed, stained, and prepared for absorbance measuring according to the method described previously (Chaieb et al. 2007). Strains were tested in at least two independent tests, each with 12 replicates. The results were interpreted as the arithmetic mean of the measured values ± standard deviation. The percentage inhibition of biofilm formation was calculated according to the formula described in a study by Jadhav et al. (2013).

Percentage inhibition = [1 − (A_E_/A_S_)] × 100.

A_E_ represents the absorbance of the well with the test strain with the tested Ent, and A_S_ represents the absorbance of the well with the test strain without Ent.

## Results

Among 14 identified *S. aureus* strains (Simonová et al. 2007), 13 MRSA were resistant to methicillin (except SA2A/3), and 5 strains (SA2A/3, SA3A/2, SA3A/3, SABel1, SAK1/2) were susceptible to amoxicillin and cefoxitin. In the remaining 8 *S. aureus* strains, the PBP2´ latex agglutination method repeatedly confirmed the phenotypic methicillin/oxacillin/cefoxitin-resistance.

Testing the enzymatic activity of selected MRSA strains, all strains showed slight production (5–10 nmol) of the most enzymes (Table 1). The majority of strains showed a positive reaction for esterase (C4; 20 nmol; except the SA2A/2 strain; 10 nmol). It was important to check if tested strains had negative reactions to β-glucuronidase and β-glucosidase; surprisingly, our strains showed only slight reactions. The strain SA6A/2 possessed the highest pathogenicity potential because of the highest values of enzymes produced compared with other tested strains.

**Table 1:** The enzymatic acitivity (nmol) of MRSA strains

MRSA	alcalic phosphatase	esterase (C4)	esterase lipase	lipase	leucin-arylamidase	Valin-arylamidase	cystin-arylamidase	trypsin	α-chymotrypsin	
SA2A/2	5	10	10	5	10	5	5	5	5	
SA5A/2	5	20	10	5	5	5	5	5	5	
SA5B/1	5	20	10	5	5	5	5	5	5	
SA6A/1	5	20	10	5	5	5	5	5	5	
SA6A/2	5	20	10	5	10	10	5	5	5	
SANip	5	20	10	5	5	5	5	5	5	
SARUM1	10	20	10	5	5	5	5	5	5	
SAK/2	5	10	10	5	5	5	5	5	5	
MRSA	acidic phosphatase	Naftol-AS-BI-phospho-hydrolase	α-galactosidase	β-galactosidase	β-glucuronidase	α-glucosidase	β -glucosidase	N-acetyl-β-glucosamonidase	α-manosidase	α-fucosidase
SA2A/2	10	10	5	5	5	5	5	5	5	5
SA5A/2	10	10	5	5	5	5	5	5	5	5
SA5B/1	10	10	5	5	5	5	5	5	5	5
SA6A/1	5	10	5	5	5	5	5	5	5	5
SA6A/2	10	10	5	5	5	10	5	5	5	5
SANip	5	10	5	5	5	5	5	5	5	5
SARUM1	5	10	5	5	5	5	5	5	5	5
SAK/2	10	10	5	5	5	10	5	5	5	5

MRSA – methicillin-resistant *S. aureus*

The majority of MRSA (6) showed β-hemolysis (Table 2); the SA2A/2 and SA6A/1 strains showed γ-hemolysis (which means they did not form hemolysis). While strains SA2A/2 and SA6A/2 did not produce the enzyme DNase, a virulence factor that catalyzes DNA degradation, the rest of the staphylococci were DNase-positive (Table 2).

**Table 2:** PBP2´ test, hemolysis, DNase activity, slime production (biofilm formation on CRA), biofilm formation (plate assay) of MRSA strains

MRSA	PBP2´test	Hemolysis	DNase activity	Biofilm formation (qualitative/quantitative)			
				CRA-24 h	CRA-48 h	CRA-72 h	Plate assay (A_570_)
SA2A/2	+	γ	-	+	+	+	0.546 (++)
SA5A/2	+	β	+	+	+	+	0.606 (++)
SA5B/1	+	β	+	+	+	+	0.556 (++)
SA6A/1	+	γ	+	+	+	+	0.556 (++)
SA6A/2	+	β	-	-	+	+	0.526 (++)
SANip	+	β	+	-	+	+	0.546 (++)
SARUM1	+	β	+	+	+	+	0.586 (++)
SAK/2	+	β	+	-	-	+	0.606 (++)

MRSA – methicillin-resistant *S. aureus*; CRA – Congo red agar, ++ - moderate biofilm formation

Testing the qualitative biofilm formation, 5 strains were positive already after 24 h, 7 strains after 48 h/72 h, and the SAK/2 strain form biofilm only after 72 h. Using the quantitative plate assay, those 7 MRSA with positive biofilm formation on CRA were also found to be positive and showed low-grade biofilm formation according to Chaieb et al. (2007; 0.1 < OD570 ≤ 1.0; Table 2). Moderate biofilm formation (0.4 < OD ≤ 0.8; ++) was observed in all strains.

The antibacterial effect of tested Ents was noted, whereas all MRSA were susceptible to tested Ents, except EntCCM4231, which was not able to inhibit the growth of all strains (Table 3). Strains SA5A/2, SA5B/1, and SA6A/2 were more resistant to Ents (100–200 AU/mL), and the 5B/1 strain did not show any inhibition zone against Ent7420 (0 AU/mL). Other strains showed higher susceptibility to tested Ents in the range of 3200–12,800 AU/mL.

**Table 3:** Antibacterial (AU/mL) of enterocins (Ent) against MRSA

MRSA	EntA/P	EntM	Ent7420	Ent9296	Ent55	Ent412	Ent4231	DurED26E/7
SA2A/2	12,800	6400	3200	3200	800	1600	0	800
SA5A/2	200	100	100	200	200	100	0	200
SA5B/1	200	100	0	100	100	100	0	100
SA6A/1	12,800	6400	12,800	6400	3200	6400	0	800
SA6A/2	400	6400	12,800	400	400	200	0	200
SANip	12,800	6400	3200	1600	1600	800	0	400
SARUM1	12,800	6400	12,800	6400	1600	3200	0	1600
SAK/2	12,800	6400	12,800	6400	3200	3200	0	1600

MRSA – methicillin-resistant *S. aureus*, Ent – enterocin, Dur - Durancin

Evaluating the antibiofilm activity/effect of the tested Ents, only Ent4231 did not inhibit the biofilm formation of MRSA strains (Table 4). The rest of the Ents exhibited a high level of antibiofilm activity, ranging from 64.9 to 97.0%. The highest antibiofilm activity, with an average of 96.4%, ranging from 84.8 to 97.0%, was shown by Ent7420. The most inhibited biofilm formation was noted in SAK/2 and SA5A/2 (92.8 respectively 92.7% on average) after Ents addition. The biofilm formed by the SARUM1 strain appeared to be the strongest, with the lowest antibiofilm activity of the tested Ents (87.5% on average).

**Table 4:** Percentage biofilm inhibition (%) exhibited by enterocins (Ents)

MRSA	EntA/P	EntM	Ent7420	Ent9296	Ent55	Ent412	Ent4231	DurED26E/7
SA2A/2	87.6	88.9	96.8	90.3	96.1	90.4	0	94.7
SA5A/2	91.0	85.7	97.0	95.9	96.9	88.2	0	94.2
SA5B/1	86.9	88.4	96.4	87.0	94.5	90.4	0	93.4
SA6A/1	88.0	86.9	96.0	87.0	96.4	88.4	0	93.2
SA6A/2	89.5	81.9	94.8	93.6	95.2	90.7	0	93.4
SANip	90.5	88.0	96.8	87.1	94.5	90.7	0	95.7
SARUM1	91.2	84.7	96.8	91.1	64.9	90.4	0	93.2
SAK/2	91.1	87.0	96.2	96.3	93.2	90.9	0	94.7
Average	89.5	86.4	96.4	91.0	91.5	90.0	0	94.1

MRSA – methicillin-resistant *S. aureus*, Ent – enterocin, Dur - Durancin

## Discussion

*Staphylococcus aureus* is a well-known commensal (skin, mucose of the respiratory, alimentary, and urogenital tract) and pathogen of a lot of animal species and humans. They are easily spread between animals and humans by skin-to-skin contact, excretions, aerosols, and animal products (meat, non-pasteurized milk), and when they are getting deeper into the organism (e.g., to blood, lungs, gastrointestinal tract, kidneys, etc.), they become pathogenic staphylococci. *S. aureus* produces a large repertoire of virulence factors, including surface-associated proteins and polysaccharides, toxins, and exoenzymes, which contribute to its success as a pathogen (Lowy 1998). The gut microbiota in the large intestine of humans and animals exhibits a variety of enzymatic activities with potential impact on the organism´s health through the biotransformation of secondary plant products and xenobiotic compounds. Some of these enzymes, such as α-chymotrypsin, β-glucuronidase, β-glucosidase, and N-acetyl-β-glucosaminidase, respectively in the presence of bacterial strains characterized by their high activity are associated with intestinal diseases and tumors (De Preter et al. 2008). Our *S. aureus* strains did not show a reaction to “negative” enzymes; however, they showed high resistance to methicillin and other tested antibiotics (Simonová et al. 2007). Although staphylococci are also known as producers of microbial lipases and esterases for their use as catalysts in the cosmetic, medicinal, food, or detergent industries (Sarmah et al. 2018), our isolates possessed only slight lipase and higher, but still moderate esterase production.

Determination of the prevalence of phenotypic virulence factors among the tested MRSA revealed that the majority of them (75%) were characterized by the presence of DNase, but still at a lower level than in *S. aureus* strains of milk origin (Gündoğan et al. 2006). Staphylococcal nuclease is an endo- and exo-nuclease that breaks down DNA and RNA substrates and it is encoded by two staphylococcal nuclease genes, Nuc (SA0746) and Nuc2 (SA1160). Kiedrowski et al. (2011) observed a correlation between Nuc activity and biofilm formation. The authors noted that Nuc levels have a significant impact on in vitro biofilm formation in *S. aureus* and can disperse biofilm by breaking down extracellular DNA. They also presented enhanced biofilm formation in strains that do not produce Nuc, which was not confirmed by us.

The presence of β-hemolysin enhances *S. aureus* colonization of the skin and can also contribute to biofilm formation (Tam and Torres 2019); a positive correlation between hemolysis and biofilm-forming ability was also noted by us. Regarding the detection of hemolytic activity, almost all MRSA showed β-hemolysis, contrary to Motamedi et al. (2018), who detected hlb genes for β-hemolysin production only in 5% of MRSA.

As it was mentioned above, *S. aureus* strains have a wide spectrum of virulence factors, including secreted and cell surface-associated factors, and especially the last of them is connected with biofilm formation. Staphylococcal bacterial biofilms remain a serious clinical problem due to resistance to antibiotics and disinfectants, as well as resistance to phagocytosis and the host immune system, and to the continued difficulties in treating staphylococcal biofilm-associated infections.

Slime factor is a viscous extracellular polysaccharidic layer that makes phagocytosis difficult and enhances adhesion to host tissues and plastic or metallic surfaces. Our MRSA strains showed a higher prevalence of biofilm-forming activity right after 24 h of testing (5 positive from 8 tested strains) and all positive after 72 h, than was presented by Dubravka et al. (2010) in *S. aureus* isolated from bovine mastitis.

Testing for biofilm production showed that all MRSA strains were moderate biofilm producers. Silva et al. (2022) also presented the ability to form biofilms by *S. aureus* isolated from a wide range of animals. Moreover, they noted an association between biofilm formation and antimicrobial resistance, with stronger biofilms produced by MDSA strains. Similar to our results, Bino et al. (2019) also detected most coagulase-negative staphylococci from horses as biofilm-forming strains. On the other hand, wild staphylococci isolated from roe deer were low-grade biofilm producers or non-forming biofilm (David et al. 2017), although two strains of them were multi-resistant to antibiotics. Pathogenic staphylococci beyond their ability to form biofilm have an amazing capacity to acquire resistance traits to antibiotics, and therefore, infections caused by these bacteria are very difficult to treat. All the tested strains that were able to form biofilm were also methicillin-resistant; the results indicate that these strains could be virulent agents.

For treating infections by multiresistant bacteria, including MDSA and MRSA, new natural compounds are needed. The synergistic effect of enterocins DD28 and DD93 (in the range of 800–6400 AU/mL of their activity) in combination with erythromycin or kanamycin against the clinical MRSA-S1 strain was noted (Al Atya et al. 2016). An equally effective and promising way to inhibit highly virulent *S. aureus* strains, including biofilm-forming and/or MRSA and MDSA, could be the application of antimicrobial peptides-bacteriocins themselves-without antibiotics. David et al. (2017) also reported the inhibition activity of partially purified enterocin PPE E3 from *E. faecalis* against *S. aureus* isolated from a wound and also a good healing process after its application right to the wound. The inhibition activity of PPEs produced by enterococci against the target of indicators, including *S. aureus* strains, has already been described (Simonová and Lauková 2004) and repeatedly confirmed in this study against MRSA. The anti-staphylococcal effect of durancin DurED26E/7 was observed, similarly to Hanchi et al. (2017), who described the high inhibition effect of durancin 61 A alone and combined with other bacteriocins and antibiotics against clinical pathogens, including MRSA.

While the majority of studies with bacteriocins used to target biofilms have used lantibiotics, other groups of bacteriocins have also been investigated. Caballero Gómez et al. (2013) and Al Atya et al. (2016) reported the efficacy of enterocins AS-48, resp. DD93 and DD28 in combination with several biocides and antibiotics against MRSA biofilms. Molham et al. (2021) observed significant destruction (80% and 48%) of *Streptococcus mutans* ATCC 25,175–associated preformed biofilms treated with crude supernatants of *E. faecium* FM43 and *E. faecium* FM50. Our tested Ents, in addition to their strong anti-MRSA activity, were also able to inhibit MRSA biofilms in a higher range (89.5–96.4%). Since studies on the direct effect of enterocins on biofilms formed by MRSA are limited, the high antibiofilm activity of tested Ents is very promising. These results show that antimicrobial peptides – enterocins - may serve as a potential treatment against biofilm-forming MDSA and MRSA strains. Further studies are needed to assess the efficacy of enterocins mostly in animal models - in vivo experiments to achieve more information regarding the most effective concentration, dose, application form, combinations, etc. of tested postbiotics.

## Conclusion

In conclusion, the tested Ents showed high antimicrobial and antibiofilm activity against MRSA strains isolated from rabbits. These bioactive substances offer a promising option for the treatment of infections caused by MRSA, with a focus on the strains with biofilm-forming ability. Justification for other laboratory and clinical studies of them is required. The advantage of Ents that they mitigate the development of bacterial resistance and biofilm formation by resistant bacterial strains. However, more significant results can be achieved by combining several bioactive substances, such as two enterocins or enterocins with another natural compounds. Therefore, Ents are promising as a candidate for the development of antibacterial drugs against MRSA.

## Data Availability

All data generated or analyzed during this study are included in this article.

## Abbreviations

MR: Methicillin resistance
MRSA: Methicillin-resistant *Staphylococcus aureus*
MDSA: Multidrug-resistant *Staphylococcus aureus*.
MRS: Methicillin-resistant staphylococci
PBP2: Penicillin-binding protein 2
CCM: Czech Culture Collection of Microorganisms
Ent: Enterocin
Dur: Durancin
PPE: Partially purified enterocin
